# Apoptotic caspase inhibits innate immune signaling by cleaving NF-κBs in both Mammals and Flies

**DOI:** 10.1038/s41419-022-05156-2

**Published:** 2022-08-24

**Authors:** Di Wu, Zhaowei Wang, Jing Zhang, Adam G. Robinson, Bao Lyu, Ziyu Chen, Chong Wang, Bin Wei, Xiaojun Xia, Qing Zhang, Xi Zhou

**Affiliations:** 1 State Key Laboratory of Virology, Wuhan Institute of Virology, Chinese Academy of Sciences, Wuhan, Hubei 430071 China; 2 State Key Laboratory of Biocontrol, School of Ecology, Sun Yat-sen University, Shenzhen, Guangdong 518107 China; 3 Lineberger Comprehensive Cancer Center, University of North Carolina School of Medicine, Chapel Hill, NC 27599 USA; 4 Department of Pathology and Laboratory Medicine, University of North Carolina, Chapel Hill, NC 27599 USA; 5 University of Chinese Academy of Sciences, Beijing, 100049 China; 6 State Key Laboratory of Oncology in South China, Collaborative Innovation Center for Cancer Medicine, Sun Yat-sen University Cancer Center, Guangzhou, Guangdong 510060 China; 7 Department of Pharmacology, University of North Carolina, Chapel Hill, NC 27599 USA

**Keywords:** Immunology, Innate immunity

## Abstract

Host organisms use different innate immune mechanisms to defend against pathogenic infections, while tight control of innate immunity is essential for proper immune induction and balance. Here, we reported that apoptotic induction or caspase-3 overexpression caused dramatic reduction of differently triggered cytokine signalings in human cells, murine primary cells and mouse model, while the loss of caspase-3 or inhibiting apoptosis markedly enhances these immune signalings. Furthermore, caspase-3 can mediate the cleavage of NF-κB members p65/RelA, RelB, and c-Rel via its protease activity. And the caspase-3-resistant p65/RelA, RelB, or c-Rel mutant mostly restored the caspase-3-induced suppression of cytokine production. Interestingly, we further uncovered that apoptotic induction also dramatically inhibited Toll immune signaling in *Drosophila*, and the *Drosophila* effector caspases, drICE and DCP-1, also mediated the degradation of DIF, the NF-κB of Toll signaling. Together, our findings demonstrate apoptotic effector caspases, including mammalian caspase-3 and fly drICE/DCP-1, can function as repressors of NF-κB-mediated innate immune signalings.

## Introduction

Host organisms perpetually face the threats of pathogens, such as bacteria, fungi and viruses, while innate immune signaling pathways serve as the countermeasures to detect and eradicate diverse pathogens [1]. A common nature of innate immunity is that various pathogen-associated molecular patterns (PAMPs), such as pathogen-derived nucleic acids, are recognized by distinct pattern recognition receptors (PRRs), which trigger a series of signaling cascades that activate canonical inhibitor of κB kinases (IKKs), IKKα and IKKβ, and the noncanonical IKKs, TANK-binding kinase (TBK1) and IKKε, leading to the activation of transcriptional factors NF-κB and interferon (IFN) regulatory factor 3 (IRF3), respectively [2, 3]. Upon activation by IKKs, NF-κBs and IRF3 translocate into nucleus, resulting in the transcriptional induction of proinflammatory cytokines and type I IFNs (IFN-I), which establish anti-pathogenic states in hosts [4, 5]. Besides, innate immune signalings also play critical anti-tumor roles in mammals [6]. On the other hand, uncontrolled immune responses are detrimental, as chronic infection and inflammation can cause autoimmune diseases and cancer [7, 8]. Thus, the tight regulation of innate immune signalings is pivotal to maintain proper immune response and balance in hosts.

Apoptosis is a major type of programmed cell death that is conserved throughout evolution, pivotal for the development and homeostasis of multicellular organisms, and also considered as an efficient antiviral defense by removing infected cells [9, 10]. Apoptosis relies on a set of cysteinyl aspartate proteases (caspases), and can be activated through either extrinsic or intrinsic pathway. The extrinsic pathway requires external stimulation that activates death receptors, resulting in the activation of initiator caspases-8 and -10, and subsequent activation of effector caspases-3 and -7 (or drICE and DCP-1 in insect). The intrinsic (or mitochondrial) pathway is activated by cellular stresses such as DNA damage, endoplasmic reticulum stress, and cytokine deprivation, which trigger mitochondrial outer membrane permeabilization (MOMP). Following MOMP, cytochrome *c* is released from mitochondria into cytosol, which induces apoptosome formation and caspase-9 activation, resulting in the activation of effector caspases-3 and -7 [11, 12].

Innate immune signalings exhibit complex regulations on apoptosis. It has been reported that NF-κB can inhibit tumor necrosis factor (TNF)-induced apoptosis [13, 14], and induce the expression of anti-apoptotic proteins such as cellular inhibitors of apoptosis 1 and 2 (c-IAP1 and c-IAP2), X-linked IAP (XIAP), etc. [15, 16]. Although NF-κB is generally considered as anti-apoptotic, it can also be pro-apoptotic in certain circumstances [17] by transcriptionally upregulating a number of pro-apoptotic proteins including p53 and Bax, or repressing some anti-apoptotic genes [17, 18]. Besides, IFN-I and IRF3 can induce the expression of multiple pro-apoptotic proteins [19], and IRF3 can directly interact with Bax, leading to MOMP and intrinsic apoptosis [20, 21].

On the other hand, apoptosis generally executes in an immunologically silent manner [22], while apoptosis defects have been linked to autoimmune diseases [22, 23]. Several recent studies reported that MOMP could induce cGAS/STING-dependent activation of IFN-I and pro-inflammatory NF-κB signaling only under caspase-deficient conditions [24–26]. And Ning et al. recently reported that caspase-3 can cleave cGAS, MAVS and IRF3 [27]. However, a large spectrum of mechanisms of how innate immune pathways, particularly NF-κB signalings, are negatively regulated by apoptosis remain to be fully addressed. Here, we uncovered that apoptotic induction or caspase-3 overexpression caused dramatic reduction of differently triggered cytokine signalings, while the loss of caspase-3 or inhibiting apoptosis markedly enhances these immune signalings both in vitro and in vivo. We further found that caspase-3 can mediate the cleavage of NF-κB members p65/RelA, RelB, and c-Rel via its protease activity, and the caspase-3-resistant p65/RelA, RelB, or c-Rel mutant mostly restored the caspase-3-induced suppression of cytokine production. Interestingly, apoptotic induction also dramatically inhibited Toll immune signaling in *Drosophila*, and similar with their mammalian ortholog, the *Drosophila* effector caspases, drICE and DCP-1, mediated the cleavage of DIF, the NF-κB of Toll signaling. These findings demonstrate apoptotic effector caspases can repress NF-κB-mediated innate immune signalings, and this regulatory mechanism is evolutionarily conserved from insects to mammals.

## Results

### Apoptosis inhibit cytokine signaling pathway

**Fig. 1:**
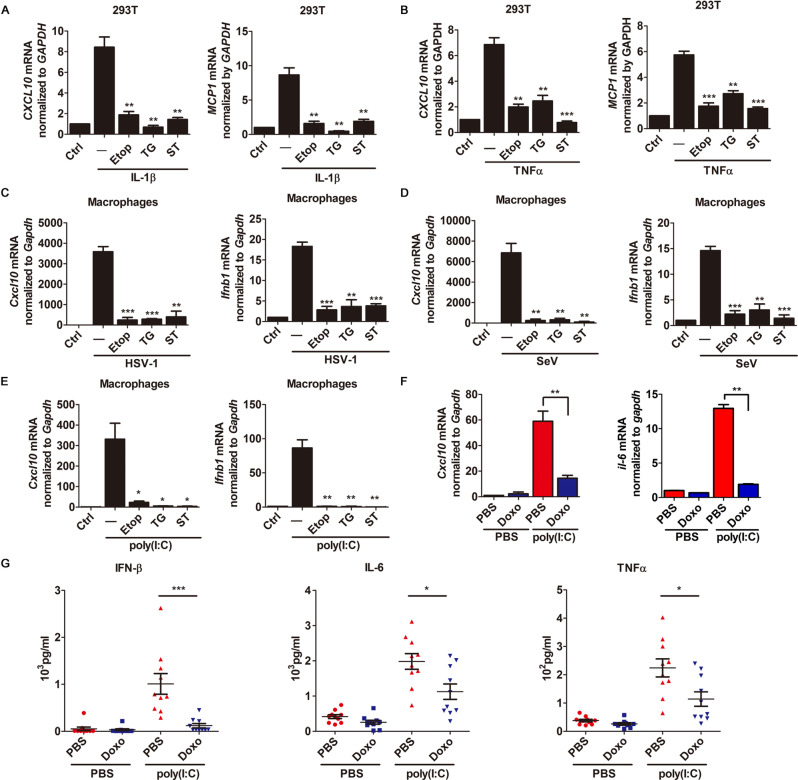
Apoptosis inhibits cytokine production. **A**, **B** Quantitative RT-PCR analysis of *CXCL10* and *MCP1* mRNAs in 293 T cells or 293-TLR3 cells treated with Etop, TG, or ST in the absence or presence of IL-1β (10 ng/mL) for 4 hr (**A**), TNFα (10 ng/ml) for 2 h (**B**). **C**, **D** Quantitative RT-PCR analysis of *Cxcl10* and *Ifnb1* mRNA in macrophages from WT mice treated with Etop, TG and ST, and infected with HSV or SeV for 8 h. **E** Quantitative RT-PCR analysis of *ifnb1*, *cxcl10* mRNA in peritoneal macrophages from WT mice treated with Etoposide (Etop, 100 μM, 24 h), Thapsigargin (TG, 2 μM, 24 h) and Staurosporine (ST, 1 μM, 8 h), then treated with poly(I:C) (2 μg/ml, 2 h). **F**, **G** Sex- and age-matched mice (*n* = 10) were ip injected of 20 mg/kg doxorubicin (DOX or Doxo) or PBS for 5 days, ip injected with poly(I:C) or PBS for 2 h. *Cxcl10* and *il-6* mRNAs of heart were analyzed by qRT-PCR (**F**), serum concentrations of the indicated cytokines were measured by ELISA (**G**). Data are representative of three independent experiments (means with SEMs). *p < 0.05, **p < 0.01, and ***p < 0.001.

Previous studies have revealed that apoptosis generally executes in an immunologically silent manner [22]. We aimed to determine the effect of apoptotic induction by using a set of canonical apoptosis inducers, including etoposide (Etop), thapsigargin (TG), and staurosporine (ST), whose apoptosis and caspase inducing activities were also confirmed (Fig. EV1A–C). To determine if apoptosis is involved in the activation of NF-κB-mediated cytokine production, we treated HEK293T cells with interleukin (IL)-1β or TNFα together with apoptosis inducer Etop, TG, or staurosporine (ST). Our results show that the induction of apoptosis potently inhibited IL-1β- or TNFα-induced transcription of NFκB-responsive cytokine genes such as *CXCL10*, monocyte chemotactic protein-1 (*MCP-1*), and *IKB*A (Fig. 1A, B; Fig. EV2A–C). We further examined if apoptosis can also suppress virus-triggered antiviral immune responses. To this end, we infected primary mouse peritoneal macrophages with Herpes Simplex Virus-1 (HSV-1) or Sendai virus (SeV) in the presence or absence of apoptotic induction. Results in Fig. 1C, D show that apoptosis significantly inhibited HSV-1- or SeV-triggered expression of *Cxcl10* and *Ifnb1*. We then treated primary mouse peritoneal macrophages with poly(I:C) in the presence or absence of apoptosis inducer, and found that apoptotic induction also dramatically inhibited poly(I:C)-induced expression of *Cxcl10* and *Ifnb1* (Fig. 1E). Moreover, the induction of apoptosis potently inhibited poly(I:C)-induced transcription of IRF3-responsive genes including *IFNB1*, *ISG15*, and *RANTES* in 293-TLR3 cells (Fig. EV2D).

To further determine the role of apoptosis in vivo, sex- and age-matched group of C57BL/6 mice were subjected to intraperitoneal (ip) injection of doxorubicin (Doxo), a potent apoptosis inducer frequently used to activate apoptosis in vivo [28], or mock treatment (i.e. PBS), in the presence or absence of poly(I:C) treatment. Our results show that the induction of apoptosis in mice significantly inhibited poly(I:C)-induced transcription of *Cxcl10*, *Il-6*, *Ifnb1*, *Isg15*, and *Rantes* in mouse hearts and the levels of IFNβ, IL-6, and TNFα in sera, as measured by the qRT-PCR and ELISA analyses, respectively (Figs. 1F, G and EV2E).

### Caspase-3 downregulates cytokine signaling pathway

**Fig. 2:**
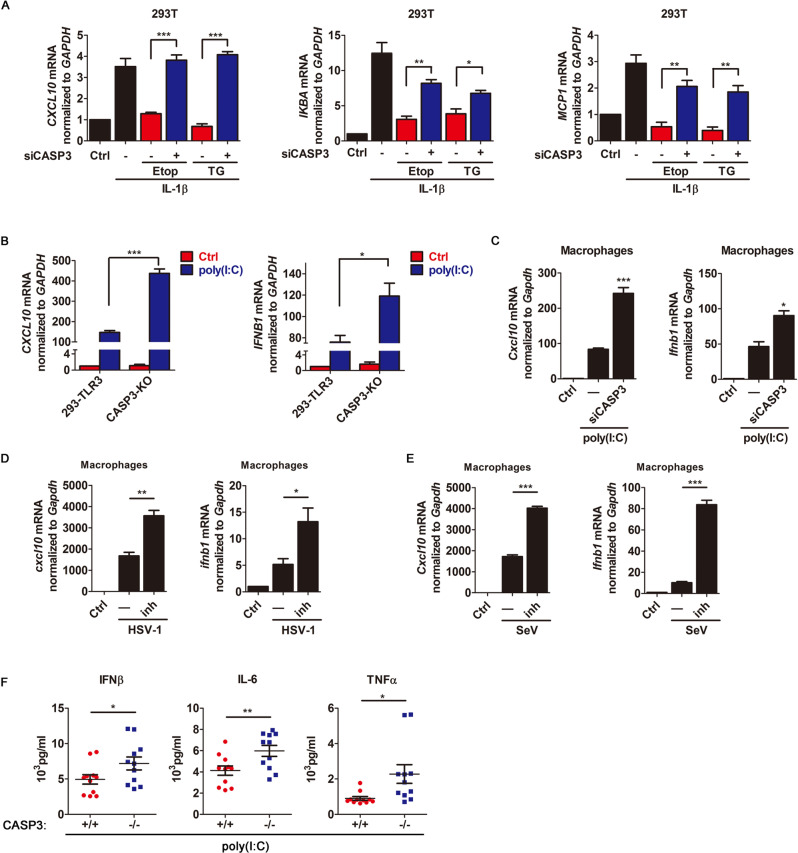
Caspase-3 downregulates cytokine production. **A** Quantitative RT-PCR analysis of *CXCL10*, *IKBA* and *MCP1* mRNA in 293 T cells transfected with CASP3-specific siRNA (siCASP3) and treated with IL-1β (10 ng/mL) for 4 h in the presence or absence of Etop and TG as indicated. **B** Quantitative RT-PCR analysis of *CXCL10* and *IFNB1* mRNA in 293-TLR3 and CASP3-KO 293-TLR3 cells treated with or without poly(I:C). **C** Quantitative RT-PCR analysis of *Cxcl10* and *Ifnb1* mRNA in primary mouse peritoneal macrophages transfected with control siRNA or indicated siRNA in the absence or presence of poly(I:C). **D**, **E** Quantitative RT-PCR analysis of *Cxcl10* and *Ifnb1* mRNA in primary mouse peritoneal macrophages treated with or without z-VAD-FMK and infected with HSV (**D**) or SeV (**E**) for 8 h. **F** CASP3^+/+^ and CASP3^−/−^ mice were injected with poly(I:C) for 2 h. Serum concentrations of the indicated cytokines were measured by ELISA. Data are representative of three independent experiments (means with SEMs). *p < 0.05, **p < 0.01, and ***p < 0.001.

Caspase-3 is the major apoptotic effector caspase that mediates the cleavage of multiple cellular substrates and execute cell death. Therefore, to determining the role of caspase-3 in the effect of apoptosis on these immune signalings, we used siRNA to knockdown of caspase-3 in 293 T cells (Fig. EV3A), and the result showed that the apoptosis-mediated repression of IL-1β-induced transcription of *CXCL10*, *IKBA*, or *MCP-1* was significantly counteracted by caspase-3 knockdown (Fig. 2A). Then, we carried out caspase-3 knockout (KO) in 293T-TLR3 cells via CRISPR-Cas9 (Fig. EV3B). Our results showed that the loss of caspase-3 significantly enhanced poly(I:C)-induced expression of *CXCL10* and *IFNB1* in 293T-TLR3 cells (Fig. 2B). And the knockdown of caspase-3 by siRNA significantly elevated IL-1β-induced expression of cytokines in human 293 T cells (Fig. EV3C), as well as poly(I:C)-induced expression of *Cxcl10* and *Ifnb1* in primary mouse peritoneal macrophages derived from C57BL/6 mice (Fig. 2C). In addition, because cytokine and IFN-I pathways can function as antiviral defense in mammals [29], we examined if caspase-3 also plays negative role in virus-triggered antiviral immune responses. To this end, we infected primary mouse peritoneal macrophages with HSV-1 or SeV in the presence or absence of caspase inhibitor (z-VAD-FMK), and found that inhibition of apoptosis significantly enhanced HSV-1- or SeV-triggered expression of *ifnb1* and *cxcl10* in macrophages (Fig. 2D, E).

To further determine the role of caspase-3 in these immune responses in vivo, caspase-3-deficient (Casp3^−/−^) C57BL/6 mice and wild-type (WT) littermates were treated with poly(I:C) via ip injection. Consistent with the observations in mouse peritoneal macrophages and human cell lines, the deficiency of caspase-3 significantly increased the levels of IFNβ, IL-6, and TNFα in sera (Fig. 2F). Together, our data show that the deficiency of caspase-3 can upregulate cytokine and IFN-I pathways in human cell lines, mouse primary cells, and mouse model.

**Fig. 3:**
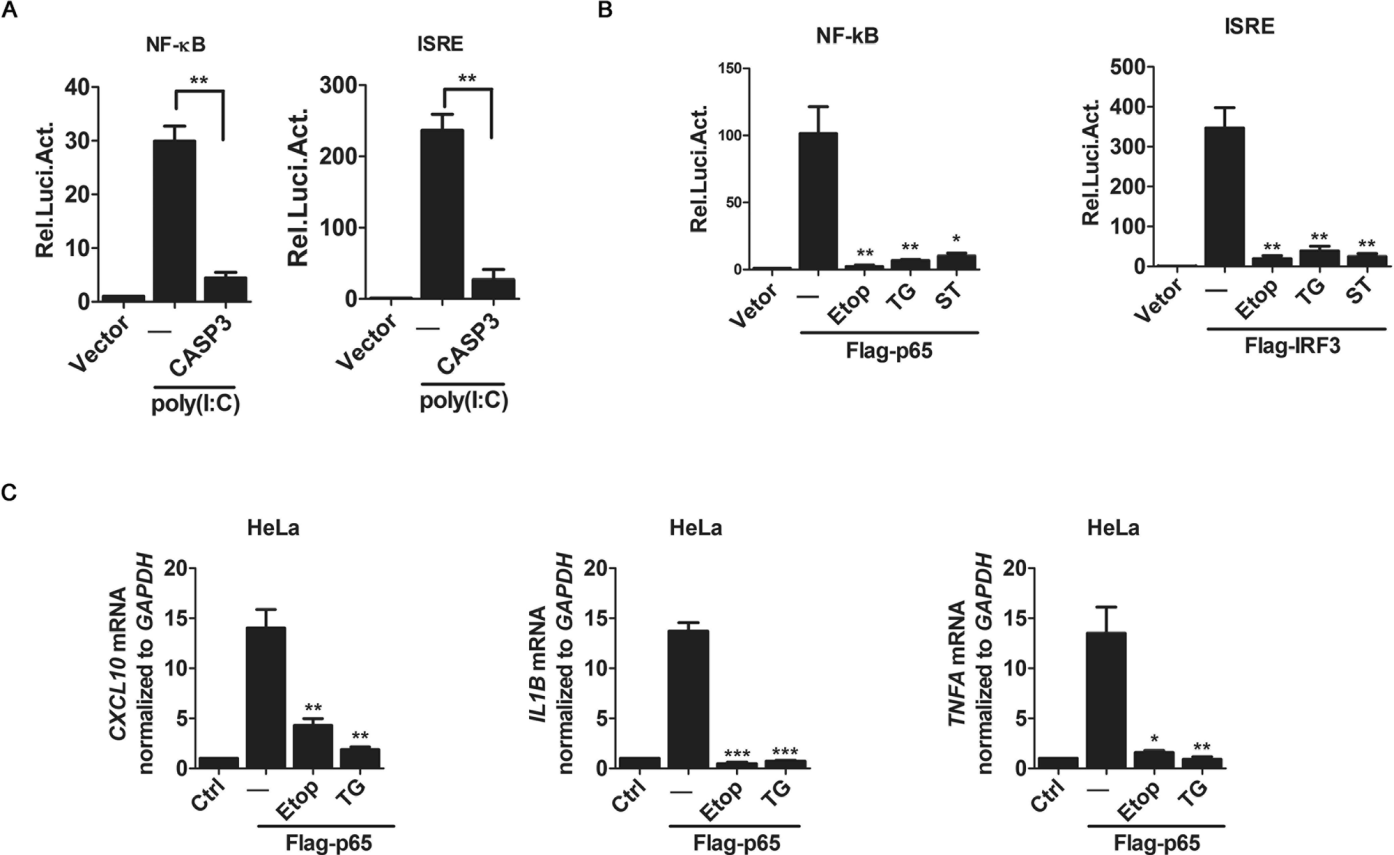
Caspase-3 downregulates cytokine production induced by p65/RelA. **A**, **B** Luciferase assay analyzing NF-κB and ISRE promoter activity in 293-TRL3 cells transfected for 48 h with the plasmids encoding NF-κB or ISRE firefly luciferase reporters and TK Renilla luciferase reporter for control, along with transfected the plasmid or treated with poly(I:C) (**A**) and Etop, TG, ST (**B**) as indicated. **C** qRT-PCR analysis of *CXCL10*, *IL1B* and *TNFA* mRNA in HeLa cells transfected with the Flag-p65 plasmid and treated with Etop or TG as indicated. Data are representative of three independent experiments (means with SEMs). *p < 0.05, **p < 0.01, and ***p < 0.001.

Then, we transfected 293-TLR3 cells with *NF-κB-luc* or *ISRE-luc* reporter DNA with or without the caspase-3 plasmid, and then treated them with poly(I:C). Our data show that the overexpression of caspase-3 potently inhibited poly(I:C)-induced NF-κB-luc and ISRE-luc activities (Fig. 3A). Moreover, we activated NF-κB signaling by overexpressing p65/RelA, and found that apoptotic induction via Etop, TG or ST dramatically inhibited p65-induced NF-κB-luc activity in the reported assay (Fig. 3B) and p65-induced cytokine production in cells (Figs. 3C and EV4A, B). In addition, consistent with the previous study [27], TBK1- or IRF3-induced ISRE-luc activities were also inhibited by apoptotic induction (Fig. 3B). These data indicate that apoptosis suppresses NF-κB signalings via caspase-3.

### Caspase-3 cleaves p65/RelA, RelB and c-Rel

**Fig. 4:**
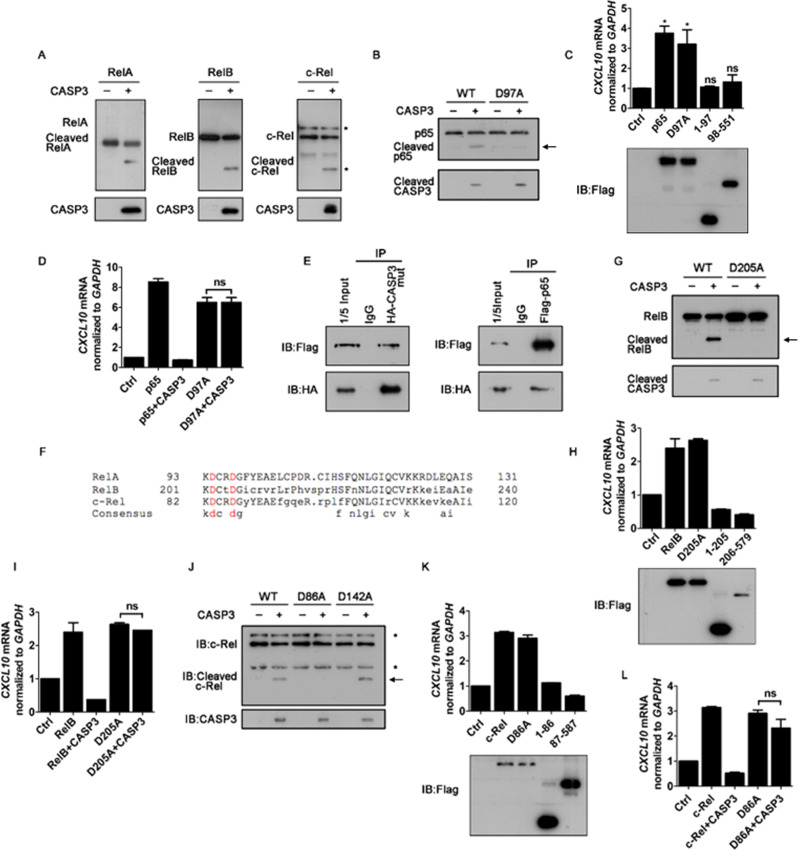
Caspase-3 cleaves RelA at D97, RelB at D205 and c-Rel at D86. **A** Immunoblot of purified N-terminal GST and C-terminal Flag tagged proteins as indicated incubated without (−) or with caspase-3 (+) for 60 min at 37 °C. The blot was probed with anti-Flag antibody. **B** Immunoblot of purified WT and D97A mutant of RelA proteins incubated without (−) or with caspase-3 (+) for 60 min at 37 °C. The blot was probed with anti-Flag antibody. **C** Quantitative RT-PCR analysis of *CXCL10* mRNA in HEK293T cells transfected with RelA and its mutants or truncations followed by immunoblot analysis. **D** Quantitative RT-PCR analysis of *CXCL10* mRNA in HEK293T cells transfected with RelA (p65), its mutant and caspase-3 as indicated. **E** 293T cells were transfected with Flag-p65 and HA-tagged protease-defective mutant CASP3. Co-immunoprecipitations were performed by using anti-HA or anti-Flag antibody, followed by immunoblot analysis with indicated antibodies. **F** Alignment of the amino acid sequence of RelA, RelB and c-Rel, the putative caspase-3 recognition motif DXXD was highlighted (red). **G** Immunoblot of purified WT and D205A mutant of RelB proteins incubated without (−) or with caspase-3 (+). The blot was probed with anti-Flag antibody. **H**, **I** Quantitative RT-PCR analysis of *CXCL10* mRNA in HEK293T cells transfected with RelB, its mutants or truncations (**H**) and caspase-3 (**I**) as indicated. **J** Immunoblot of purified WT, D86A and D205A mutant of c-Rel proteins incubated without (−) or with caspase-3 (+). The blot was probed with anti-Flag antibody. **K**, **L** Quantitative RT-PCR analysis of *CXCL10* mRNA in HEK293T cells transfected with c-Rel, its mutants or truncations (**K**) and caspase-3 (**L**) as indicated. Data are representative of three independent experiments (means with SEMs). *p < 0.05, **p < 0.01, and ***p < 0.001.

Since previous work showed that caspase-3 is able to cleave p65/RelA in vitro [13, 30, 31], we examined if caspase-3 can cleave NF-κB family proteins. To this end, we produced and purified recombinant p65/RelA, RelB and c-Rel as the fusion proteins with N-terminal GST and C-terminal Flag tags. These recombinant proteins were then incubated with purified active human recombinant caspase-3. Our data showed that caspase-3 clearly cleaved p65/RelA, RelB, and c-Rel (Fig. 4A).

Based on the molecular mass of the cleaved p65/RelA band and the typical caspase-3 recognition motif DXXD, we speculated that D97 is the putative cleavage site of p65/RelA. Our data show that the D97A mutation completely blocked caspase-3-mediated cleavage (Figs. 4B and EV5A).

It is interesting to determine if the cleaved p65/RelA fragments are inactive or not. To this end, we constructed the plasmid expressing p65/RelA 1-97 aa or 98-551 aa to mimic the cleaved p65/RelA fragments. Our data show that the two cleaved p65/RelA fragments completely lost the activity to induce *CXCL10* and *IL-8* production (Figs. 4C and EV5B), indicating that p65/RelA was inactivated by the caspase-3-mediated cleavage. On the other hand, while the D97A mutant of p65/RelA retained the full activity like wild-type (WT) p65/RelA, the cytokine production induced by p65/Rel_D97A_ was resistant to caspase-3-mediated repression (Fig. 4D), showing that the caspase-3-mediated cleavage at D97 of p65/RelA is responsible for apoptosis-mediated repression of cytokine production.

Moreover, we ectopically expressed Flag-tagged p65/RelA together with HA-tagged protease-defective caspase-3 mutant in 293 T cells, and performed co-immunoprecipitation with either anti-Flag or anti-HA antibody. Our results show that the protease-defective caspase-3 and p65/RelA could be co-immunoprecipitated (Fig. 4E), indicating that caspase-3 and p65/RelA can interact with each other.

To further investigate the effects of caspase-3 on other NF-κBs, we performed sequence alignment of the Rel family members p65/RelA, RelB, and c-Rel, and found RelB and c-Rel also have typical caspase-3 recognition motifs DXXD (Fig. 4F). Therefore, we generated the D205A mutant of RelB, and found the D205A mutation completely blocked caspase-3-mediated cleavage (Figs. 4G and EV5C), indicating D205 is RelB cleavage site. Similar with p65/RelA, the cleavage mimic fragments (aa 1-205 and 206-579) of RelB lost their NF-κB function (Figs. 4H and EV5D), while the cytokine production induced by RelB_D205A_ was resistant to caspase-3-mediated repression (Fig. 4I). Furthermore, in order to determine the caspase-3 cleavage site of c-Rel, we constructed two mutations, D86A and D142A, because c-Rel contains two DXXD motifs. Our data show that caspase-3 failed to cleave c-Rel_D86A_ but effectively cleaved RelB_D142A_, indicating that the cleavage site is D86 (Figs. 4J and EV5E). We also found that the cleavage mimic c-Rel fragments (aa 1-86 and 87-587) lost their NF-κB function and c-Rel_D86A_ activated cytokine production, which was resistant to caspase-3 (Figs. 4K, L and EV5F). Together, our findings show that caspase-3 can mediate the cleavage of p65/RelA at D^97^, RelB at D^205^ and c-Rel at D^86^, resulting in the apoptosis-mediated repression of NF-κB signalings.

### *Drosophila* drICE and DCP-1 cleave DIF

Similar with mammals, invertebrates, particularly insects, also use diverse innate immune signaling pathways, like Toll and IMD pathways, to defend against pathogenic infections. In these pathways, Toll pathway is the counterpart of mammalian TLR pathway, and can be activated by the infection of fungi, Gram^+^ bacteria, and certain viruses, leading to the activation of Toll through a cascade of extracellular proteolytic events. Activated Toll then activates DIF through a signaling cascade via dMyD88, Pelle, Tube, and Cactus (IκB), resulting in the transcriptional induction of multiple antimicrobial peptides (AMPs) including *Drosomycin* (*Drs*) [32, 33]. Given that DIF is also a member of the Rel subfamily, it would be intriguing to examine if effector caspases also inhibit insect DIF and Toll pathway.

**Fig. 5:**
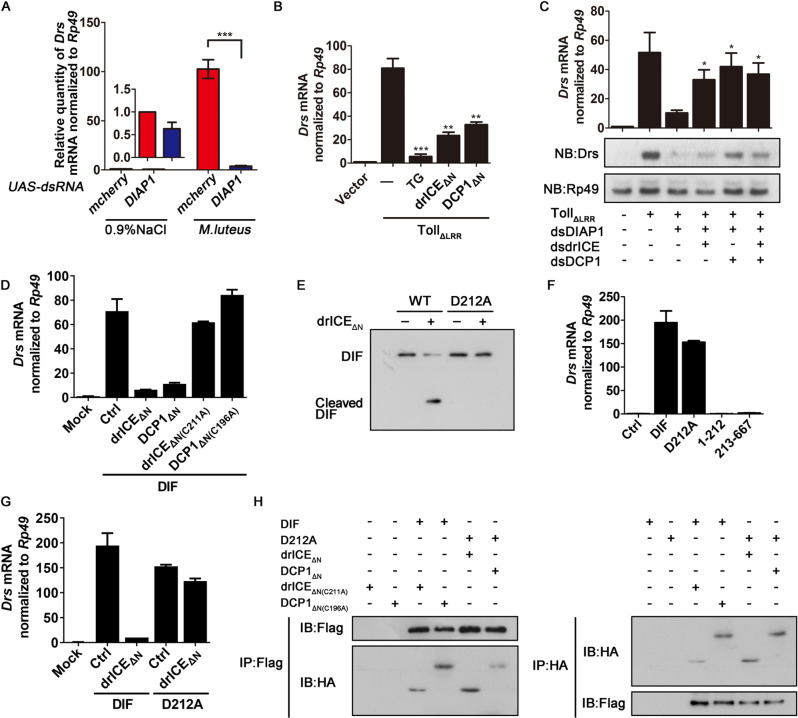
DrICE and DCP-1 cleave DIF in Drosophila. **A** At 6 h post *M. luteus* challenge, total RNA extracts were prepared from adult flies with the indicated genotypes and treatments, and subjected to qRT-PCR of Drs mRNA normalized by Rp49. Each group contains 10 female flies and 10 male flies. **B** Quantitative RT-PCR analysis of *Drs* mRNA in S2 cells transfected with HA-tagged Toll_ΔLRR_, together with drICE_ΔN_ or DCP1_ΔN_ or treatment with TG. **C** Quantitative RT-PCR analysis of *Drs* mRNA in S2 cells transfected with HA-tagged Toll_ΔLRR_ together with indicated dsRNAs. Total RNA extracts were prepared and detected via Northern blots using the indicated probes. **D** Quantitative RT-PCR analysis of *Drs* mRNA in S2 cells transfected with plasmids as indicated. **E** Immunoblot of purified WT and D212A mutant of DIF proteins incubated without (−) or with purified drICE_ΔN_ (+) for 60 min at 37 °C. The blot was probed with anti-Flag antibody. **F**, **G** Quantitative RT-PCR analysis of *Drs* mRNA in S2 cells transfected with plasmids as indicated. **H** The S2 cells were ectopically expressed with the indicated proteins for 48 h. Co-immunoprecipitation were performed by using anti-HA or anti-Flag antibody, followed by immunoblot with indicated antibodies.

In fruit flies, DIAP1, the ortholog of mammalian XIAP, serves as a central death regulator by inhibiting effector caspases drICE and DCP-1 in response to multiple stimuli [34]. To determine if apoptosis is involved in Toll signaling in vivo, we used transgenic flies carrying a DIAP1 dsRNA construct driven by the Gal4/UAS promoter. Our results showed that ubiquitous expression of Gal4 with the Actin driver in UAS-dsRNA (DIAP1) flies resulted in a successful knockdown of DIAP1 (Fig. EV6A) and a dramatic inhibition of the Toll signaling stimulated by the inoculation of *Micrococcus luteus* (*M. luteus*), a Gram^+^ bacterium (Fig. 5A), showing that apoptotic induction can inhibit Toll signaling in adult flies.

In *Drosophila*, drICE and DCP-1 are effector caspases and orthologs of caspase-3. Previous studies showed that the partial loss of leucine rich repeats (LRRs) makes Toll protein constitutively active to induce downstream signaling [35, 36]. Thus, we induced apoptosis via TG or Etop, or overexpressing N-terminally cleaved/activated drICE or DCP-1 (drICE_ΔN_ or DCP-1_ΔN_), which is constitutively active [34], in *Drosophila* S2 cells expressing Toll mutant lacking LRR (Toll_ΔLRR_). Our results showed that apoptotic induction or overexpressing drICE_ΔN_ or DCP-1_ΔN_ significantly inhibited the Toll_ΔLRR_-induced Toll signaling (i.e., the mRNA levels of *Drs* detected by qRT-PCR) (Figs. 5B and EV6B). Besides, we also induced apoptosis by knocking down *Drosophila* inhibitor of apoptosis 1 (DIAP1) (Fig. EV6C). Our data show that the repressive effect of apoptotic induction by DIAP1 knockdown on Toll_ΔLRR_-induced Toll signaling was effectively neutralized by the knockdown of drICE and/or DCP-1 (Figs. 5C and EV6D, E). These data indicate that effector caspases mediated downregulation of Toll signaling. Furthermore, our data show overexpressing drICE_ΔN_ or DCP-1_ΔN_ significantly downregulated Toll signaling induced by DIF, but protease-defective drICE_ΔN(C211A)_ or DCP-1_ΔN(C196A)_ (Fraser & Evan, 1997)_._ failed to do so (Fig. 5D).

Considering DIF belongs to the Rel subfamily, it would be intriguing to examine if drICE or DCP-1 directly cleave DIF. To this end, we performed an in vitro cleavage assay by incubating purified DIF or DIF_D212A_ and drICE_ΔN_. Our data show that the presence of drICE_ΔN_ did result in the cleavage of DIF, but D212A completely blocked drICE_ΔN_-mediated cleavage (Fig. 5E), indicating that the cleavage site is D212. To further tested if drICE_ΔN_-mediated DIF cleavage result in its inactivation, we constructed plasmids expressing DIF 1-212 aa and 213-667 aa to mimic the cleaved DIF fragments. Our data show that the cleaved DIF fragments completely lost their ability to induce *Drs* transcription (Fig. 5F), while DIF_D212A_ effectively induced Toll signaling, which was resistant to drICE_ΔN_ (Fig. 5F, G).

In addition, we examined if drICE_ΔN_ or DCP-1_ΔN_ can interact with DIF. WT or D231A Flag-DIF was ectopically expressed together with HA-tagged WT or protease-defective mutant drICE_ΔN_ or DCP-1_ΔN_ in S2 cells, followed by co-immunoprecipitation using anti-Flag or anti-HA antibody. Our results show that DIF_WT_ interacted with protease-defective drICE_ΔN_ or DCP-1_ΔN_, while DIF_D212A_ interacted with WT drICE_ΔN_ or DCP-1_ΔN_ (Fig. 5H). In conclusion, our findings show that effector caspases drICE or DCP-1 can interact with and cleave DIF.

## Discussion

**Fig. 6:**
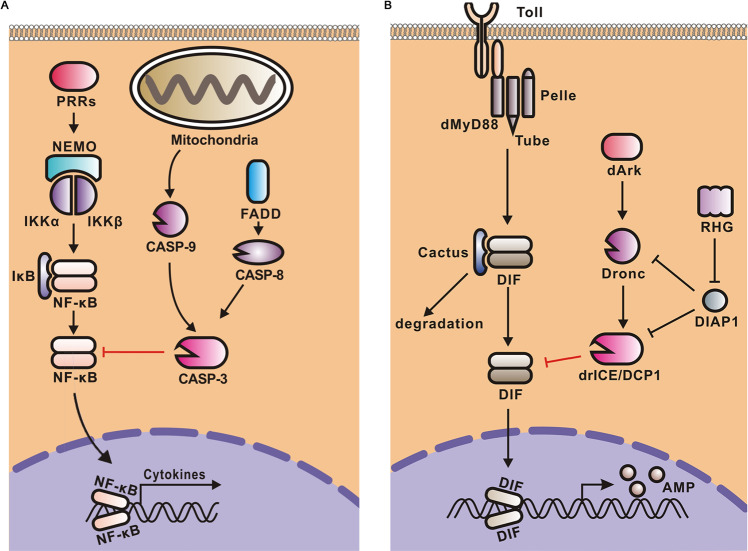
Models for the negative regulation of NF-κB signaling pathways by caspase-3-mediated cleavage of NF-κBs. **A** In mammals, apoptotic effector caspase-3 cleaves of NF-κB members p65/Rel A, RelB, and c-Rel resulting in a comprehensive repression of both NF-κB signaling pathways, which is proposed to act as a self-protective mechanism to prevent overreactive innate immune responses. **B** In Drosophila, apoptosis mediates the proteolysis of DIF, the NF-κB of Toll pathway, resulting in the repression of Toll immune signaling, dependently of the effector caspases drICE and DCP-1.

Innate immune pathways play pivotal antiviral and anti-tumor roles in multicellular organisms, while their dysregulation often contributes to autoimmunity and cancer. Thus, better understanding the mechanisms of how innate immune signalings are tightly controlled has particular importance. In this study, we reported that apoptotic caspase-3 mediates the cleavage of NF-κB members p65/RelA, Rel B, and c-Rel, resulting in the downregulation of NF-κB signalings; and this mechanism is also conserved in fruit flies, as *Drosophila* effector caspases drICE and DCP1 mediate the cleavage and degradation of DIF (Fig. 6).

Among multiple forms of programmed or non-programmed cell death, apoptosis is generally considered as immunologically silent or less inflammatory [23]. Previous studies have provided in vitro evidence that caspase-3 can mediate proteolysis or cleavage of p65/RelA, IKKβ, and NEMO [37–39]. However, NF-κB signalings, including that involving p65/Rel A, can be activated independently of IKKβ and NEMO, and p65/Rel A is not the only NF-κB member required for NF-κB signalings [40, 41]. Considering that p65/RelA, RelB or c-Rel is involved in each functional NF-κB homo- or heterodimer, our findings demonstrate that caspase-3 can mediate a comprehensive shutoff of NF-κB-mediated cytokine pathway. Besides, *Drosophila* drICE or DCP1 can mediate the cleavage of DIF, which is also a member of the Rel subfamily of NF-κB, suggesting that the effector caspase-mediated downregulation of NF-κBs is conserved throughout evolution.

In the context of viral infection, antiviral innate immune signalings are induced to restrict viral propagation. However, in certain circumstances, virus-induced immune responses would become overreactive and persistent, thereby triggering chronic inflammatory diseases and even cancer [7, 42, 43]. Given that many viral infections also induce apoptosis, the caspase-3-mediated suppression of proinflammatory cytokine production likely provides an anti-inflammatory control to prevent detrimental immune overreaction and achieve immune homeostasis during viral infection. Interestingly, the effector caspase-dependent immune repression is evolutionarily conserved from insects to mammals. Similar with that of mammals, viral infection of insects also induces both innate immunity, like Toll signaling, and apoptosis [44]. Although insects lack adaptive immunity and have lower risk of inflammation, overreactive innate immunity can trigger adverse cellular responses such as uncontrolled autophagy and hematopoietic defect [45, 46]. Thus, the tight control of virus-induced innate immune response would also be desired in insects. Besides, considering that numerous viruses encode viral inhibitors of apoptosis (vIAPs) [9], these vIAPs, particularly the ones that can be persistently expressed in infected hosts, might act as pro-inflammatory factors and serve as ideal targets of therapeutic intervention for virally induced inflammations.

In conclusion, our findings demonstrate that apoptotic effector caspases, including mammalian caspase-3 and fly drICE/DCP-1, can act as repressors of NF-κB-mediated immune signalings, which can serve as a self-protective mechanism to prevent immune overreaction. Moreover, this regulatory mechanism is well conserved throughout evolution from insects to mammals, highlighting the physiological importance of the tight control of innate immunity by caspases. Future studies should endeavor to elucidate the detailed mechanisms of mutual regulations between apoptotic caspases and innate immunity in different physiological and pathological circumstances. In addition, based on our findings, modulations of caspases or targeting vIAPs could be promising strategies to develop novel therapies against viral infection, virally triggered chronic inflammation, and autoimmune diseases, therefore representing an exciting avenue for future studies.

## Materials and methods

### Cell culture and transfection

*Drosophila* S2 cells were obtained from China Center for Type Culture Collection (CCTCC) and maintained in Schneider’s Insect Medium (Sigma) supplemented with 10% fetal bovine serum (FBS, GIBCO) and 1% penicillin/streptomycin at 27.5 °C. HEK293T cells maintained in Minimum Essential Medium (MEM) (GIBCO) supplemented with 10% FBS at 37 °C in a humidified atmosphere with 5% CO_2_. The HEK293 cells stably expressing TLR3 (293-TLR3) were generously provided by Prof. Bo Zhong (Wuhan University, Wuhan, China).

### Reagents and antibodies

Poly(I:C) HMW (Invivogen Cat# tlrl-pic); IL-1β (PEPROTECH 200-01B Cat# tlrl-pic); Peptidoglycans (PGN) (SIGMA Cat#77140 Peptidoglycan from Staphylococcus aureus); TNF-α (PEPROTECH Cat# 300-01 A); Doxorubicin (hydrochloride) (MCE Cat#HY-15142); Staurosporine (MCE Cat#HY-15141); Thapsigargin (SIGMA Cat# T9033) Etoposide (Selleck Cat#33419-42-0); Caspase inhibitor z-VAD-FMK (Beyotime Cat#C1202); Caspase 3 inhibitor Ac-DEVD-CHO (Beyotime Cat#C1206); ELISA kit for murine IFN-β, TNFα, IL-6 (BioLegend Cat#439408, Cat#430907, Cat#431307); PARP(46D11), phospho-NF-κB p65(Ser536), Phsopho-IRF-3 (Ser396) monoclonal Antibody (CST Cat#9532, Cat#3033, Cat#4947); IRF3, TBK1, RELB, c-Rel Polyclonal Antibody (ABclonal Cat#A11118, Cat#A2573, Cat#A0519, Cat#A1181); p65 RELA polyclonal Antibody (ProteinTech Cat#10745-1-AP); p-TBK1(phosphor S172), IKKi/IKKε antibody (Abcam Cat#ab109272, Cat#ab124766); Caspase-3(E-8) (Santa Cruz Cat#sc-7272). GSTSep Glutathione Agarose Resin (Yeasen 20507ES10). Caspase-Glo 3/7 Assay (Promega G8090). Protein A/G Magnetic Beads (Bimake B23202)

### Constructs

The NF-κB and ISRE luciferase reporter plasmids were kindly provided by Prof. Yan-Yi Wang (Wuhan Institute of Virology, Wuhan, China). The mammalian expression plasmids for p65, TBK1, IRF3 and IRF7 were kindly provided by Prof. Bo Zhong (Wuhan University, Wuhan, China). Toll_ΔLRR_, DIF, DIF_D231A_, drICE_ΔN_, drICE_ΔN_(C211A), DCP1_△N_, DCP1_△N_(C196A), Rpr, and DmIKKε were cloned into *Drosophila* expression plasmid pAc5.1/V5-His, and CASP3, protease-defective mutant CASP3, and IKKε were cloned into mammalian expression plasmid pCDNA3.1. The point mutations of Flag-IRF3 were constructed by PCR-mediated site-directed mutagenesis as described previously [47]. Gene-specific primers used for plasmid construction are listed in Table S1.

### Mice

*Casp3*^*+/−*^ mice were purchased from Jackson lab (stock No: 006233). These mice were inter-crossed to obtain age and sex-matched *Casp3*^+/+^ and *Casp3*^−/−^ mice. Then, these mice were ip injected with poly (I:C) (2 μg/g, InvivoGen). Animal experiments complied with National Institute of Health guidelines and were approved by the University of North Carolina at Chapel Hill Animal Care and Use Committee.

C57BL/6 mice were purchased from Beijing Vital River Laboratory Animal Technology Co. Age and sex-matched mice were ip injected were with 20 μg/g Doxo or PBS for 5 days, followed by 2 μg/g poly(I:C) or PBS treatment for 2 h [48]. The serum was collected by centrifugation. Experimental procedures were performed according to protocols approved by the Institutional Animal Care and Use Committee of Wuhan Institute of Virology, CAS.

After treatment for 2 h, blood was collected by heart puncture in EDTA treated tubes and serum was collected by centrifugation. Cytokine levels (including TNF-α, IL-6 and IFN-β) were measured by using ELISA. ELISA kit for mouse IFN-β (Cat# 439407), TNFα (Cat# 430907), and IL-6 (Cat# 431307) were ordered from BioLegend. RNA was extracted from mice hearts and analyzed by qRT-PCR. qRT-PCR primers are listed in Table S1.

### Isolation of murine peritoneal macrophages, BMDCs and MLFs

The murine peritoneal macrophages were isolated as previously described [49]. In brief, 1 ml of 3.8% Brewer thioglycollate medium was injected into the peritoneal cavity of mouse. Three days after injection, 5 ml of cold DPBS was injected into the peritoneal cavity of mouse and the fluid was aspirated carefully without puncturing any organ. The phenotype of isolated cells was examined by flow cytometry using anti-F4/80 antibody (a surface antigen expressed on macrophages).

For induction of BMDCs, bone marrow cells were isolated from mouse femur and following cultured in RPMI 1640 (GIBCO) medium supplemented with 10% FBS, murine GM-CSF (20 ng/ml) and IL-4 (10 ng/ml). The medium was changed every other day. On day 9, cells were used for subsequent analysis.

Primary mouse lung fibroblasts (MLFs) were isolated from 8-10-week-old mice and cultured in DMEM (GIBCO) medium supplemented with 10% FBS.

### Transfection and reporter gene assays

DNA or RNA transfection was performed as previously described [47]. In brief, *Drosophila* S2 cells were plated in six-well plates with about 1 × 10^6^ cells per well and then transfected with 2 μg of plasmids or RNAs using FuGENE HD Transfection Reagent (Roche) according to the manufacturer’s protocol. For PGN challenge, the cells were challenged by Lys-PGN [extract from *S. aureus* (Sigma)] 48 h after the transfection.

HEK293T or 293-TLR3 cells were plated on 24-well plates (2 × 10^5^ cells per well) and transfected with the luciferase reporter plasmid (50 ng) together with the expression plasmid or empty control plasmid (0.5 μg each). For each transfection, we added 20 ng of pRL-TK Renilla luciferase reporter plasmid to normalize the transfection efficiency. In addition, empty vector was added to ensure that each transfection receives the same amount of total DNA. Luciferase activity in total cell lysates was measured with a dual-specific luciferase reporter assay system (Promega).

### RNA interference

For gene knockdown in S2 cells, specific dsRNAs were synthesized by in vitro transcription using T7 RNA polymerase (Promega). The siRNAs targeting human CASP3 were synthesized by RiboBio (Guangzhou, China). The primers used for dsRNA preparation and the sequences of siRNAs are shown in Table S1.

### Northern blots, RT-PCR, and qRT-PCR

Total RNAs were extracted from cells or mouse tissues using TRIzol reagent (TaKaRa Bio) and treated with RQ1 RNase-free DNase I (Promega) as previously described [50]. Northern blots were performed according to our standard procedures [47]. The DIG-UTP (Roche) labeled probes for detection of *Drosomycin* and *Rp49* mRNA were synthesized by in vitro transcription using T7 RNA polymerase. The primers used for RNA probe preparation are listed in Table S1.

For RT-PCR, RNAs were subjected to reverse transcription with All-in-One cDNA Synthesis SuperMix (Bimake) using random primers as previously described [47]. The qRT-PCR was performed using 2× SYBR Green qPCR Master Mix (Bimake) according to the manufacturer’s protocol. Gene-specific primers used for PCR amplification or qRT-PCR are listed in Table S1.

### Co-immunoprecipitation (co-IP) and Western blots

Cells were harvested and then lysed in the cell lysis buffer [20 mM Tris-HCl (pH 7.4), 200 mM NaCl, 2.5 mM MgCl_2_, 0.5% Triton X-100, 0.5 U/μL RNase inhibitor (Promega) and a protease inhibitor cocktail (Roche)]. The cell lysates were subjected to 10% SDS–polyacrylamide gel electrophoresis (PAGE) and Western blots as previously described [47].

For co-IP, HEK293T cells (5 × 10^6^) seeded on 10-cm dishes were transfected with a total of 10 μg of the indicated plasmids. At 48 h after transfection, cell lysates were incubated with the indicated antibodies at 4 °C for 12 h. Then lysates were incubated with 20 μl of Protein A + G Agarose (Beyotime) at 4 °C for 2 h. The Agarose beads were then washed three times with 1 ml of lysis buffer. The precipitates were analyzed by Western blots as described above.

### Flow cytometry and TUNEL assay

Cell death was assessed by Annexin V-FITC/PI double staining (Beyotime) following manufacturer’s instructions. After acquisition by flow cytometry (BD FACSAria), data were analyzed and imaged with FCS Express 5 Plus (De Novo Software) with adapted settings.

Detection of apoptotic cells using TUNEL staining (Roche) was performed following manufacturer’s instructions. In the same experiment, detection of DNA using DAPI staining (Sigma) was performed following manufacturer’s instructions.

### In vitro caspase cleavage assay and caspase activity assay

In vitro caspase cleavage assay was performed as previously described [51, 52]. Briefly, bacterially expressed drICE with GST tag were purified and then eluted with 10 mM Glutathione in CHAPS buffer (20 mM HEPES, pH 7.0, 10 mM KCl, 0.1% CHAPS). Flag-DIF was immunoprecipitated from S2 cell lysates, followed by elution and purification by using Flag peptide. Eluted Flag-DIF was then incubated with purified drICE in CHAPS buffer in a total 20 μl reaction at 37 °C for 60 min. The cleavage products were analyzed by SDS-PAGE and visualized by western blotting.

Caspase activity was measured using Caspase-Glo manufacturer’s instructions (Promega). Cell viability was measured using CellTiter-Blue Cell Viability kit (Promega) following the manufacturer’s instructions.

### CRISPR/Cas9 Knockout

The CASP3 CRISPR/Cas9 KO Plasmid (sc-400365, Santa Cruz Biotech) was used to knockout CASP3 gene in human 293-TLR3 cells according to the manufacturer’s protocol. Briefly, after transfection with the CRISPR/Cas9 KO plasmids, 293-TLR3 cells were allowed for cultured for 3 days and then selected by puromycin (10 μg/mL) (Invivogen). The resulting clones were confirmed by Western blotting.

### Fly stocks and microbial challenge

Adult flies were reared at 25 °C and fed a standard cornmeal/yeast diet. Adult flies were randomly allocated, and the sample size was chosen according to the previous study [50]. The flies with UAS-dsRNA (mCherry) were used as WT controls. The Actin-GAL4/CyO-PscGFP driver line was obtained as previously described [50]. The UAS-dsRNA (Diap1) (stock No:33597) and UASdsRNA(mCherry) (stock No:35787) fly lines were obtained from the Bloomington Stock Center. M. luteus was provided by Dr. Fang Peng (Wuhan University, Wuhan, China). For the *M. luteus* and *E. coli* challenge, adult flies (5 days old) were inoculated by a needle previously dipped into a concentrated culture of bacteria.

### Quantification and Statistical Analysis

GraphPad Prism was used for all statistical analyses. Statistical analysis was carried out by unpaired *t* test, mean ± SEM (GraphPad Prism). *P < 0.05, **P < 0.01, and ***P < 0.001. A *P*-value < 0.05 was considered statistically significant.

## Supplementary information

Expanded View

Original Data File

previous submission (CDDIS-22-1865R)

Reproducibility checklist

## Data Availability

All datasets generated and analysed during this study are included in this published article and its Supplementary Information files. Additional data are available from the corresponding author on reasonable request.
