# Supplementary material for: Apoptotic caspase inhibits innate immune signaling by cleaving NF-κBs in both Mammals and Flies
Source: Cell Death Dis. 2022 Aug 24;13(8):731. doi: 10.1038/s41419-022-05156-2 (PMC9402571; doi:10.1038/s41419-022-05156-2)
Supplement: Supplementary file 1 — Expanded View [file 41419_2022_5156_MOESM1_ESM.pdf]

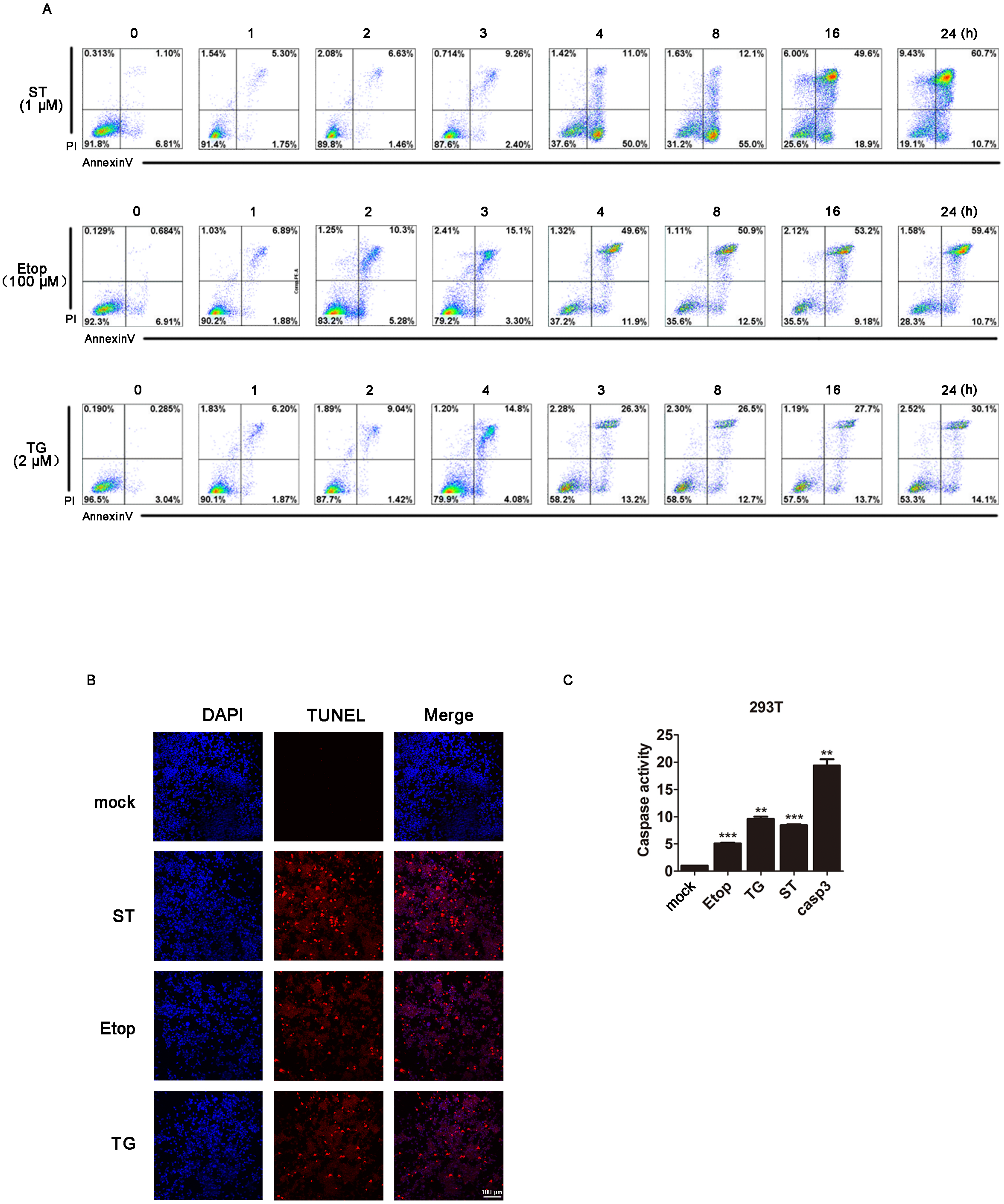
A
ST
(1 μM)
0
0.313% 1.10% 91.8% 6.81%
1
1.54% 5.30% 91.4% 1.75%
2
2.08% 6.63% 89.8% 1.46%
3
0.714% 9.26% 87.6% 2.40%
4
1.42% 11.0% 37.6% 50.0%
8
1.63% 12.1% 31.2% 55.0%
16
6.00% 49.6% 25.6% 18.9%
24 (h)
9.43% 60.7% 19.1% 10.7%
PI
AnnexinV
Etop
(100 μM)
0
0.129% 0.684% 92.3% 6.91%
1
1.03% 6.89% 90.2% 1.88%
2
1.25% 10.3% 83.2% 5.28%
3
2.41% 15.1% 79.2% 3.30%
4
1.32% 49.6% 37.2% 11.9%
8
1.11% 50.9% 35.6% 12.5%
16
2.12% 53.2% 35.5% 9.18%
24 (h)
1.58% 59.4% 28.3% 10.7%
PI
AnnexinV
TG
(2 μM)
0
0.190% 0.285% 96.5% 3.04%
1
1.83% 6.20% 90.1% 1.87%
2
1.89% 9.04% 87.7% 1.42%
4
1.20% 14.8% 79.9% 4.08%
3
2.28% 26.3% 58.2% 13.2%
8
2.30% 26.5% 58.5% 12.7%
16
1.19% 27.7% 57.5% 13.7%
24 (h)
2.52% 30.1% 53.3% 14.1%
PI
AnnexinV
B
DAPI
TUNEL
Merge
mock
ST
Etop
TG
100 μm
C
293T
Caspase activity
0
5
10
15
20
25
mock
Etop
TG
ST
casp3
***
**
***
**

Figure EV1. Etop, TG and ST induce apoptosis and caspase activity in 293T cells. Related to Figure 1

(**A**) 293T cells were treated with ST, Etop, TG for indicated time. Annexin V-FITC/PI double staining and flow cytometry assay was performed to quantify viable (Annexin V-FITC-/PI-), early apoptotic (Annexin V-FITC+/PI-) and late apoptotic cells (Annexin V-FITC+/PI+). (**B**) 293T cells were treated with ST, Etop, TG and analyzed by a TUNEL assay. Detection of DNA using DAPI staining was performed in the same experiment. TUNEL+ signals are red and DAPI+ signals are blue. (**C**) 293T cells were transfected with empty vector or vector for HA-caspase-3, and treated with or without Etop, TG, or ST. The relative caspase activity was determined, and normalized to cell viability.

Data are representative of three independent experiments (means with SEMs). $*p < 0.05$, $**p < 0.01$, and $***p < 0.001$.

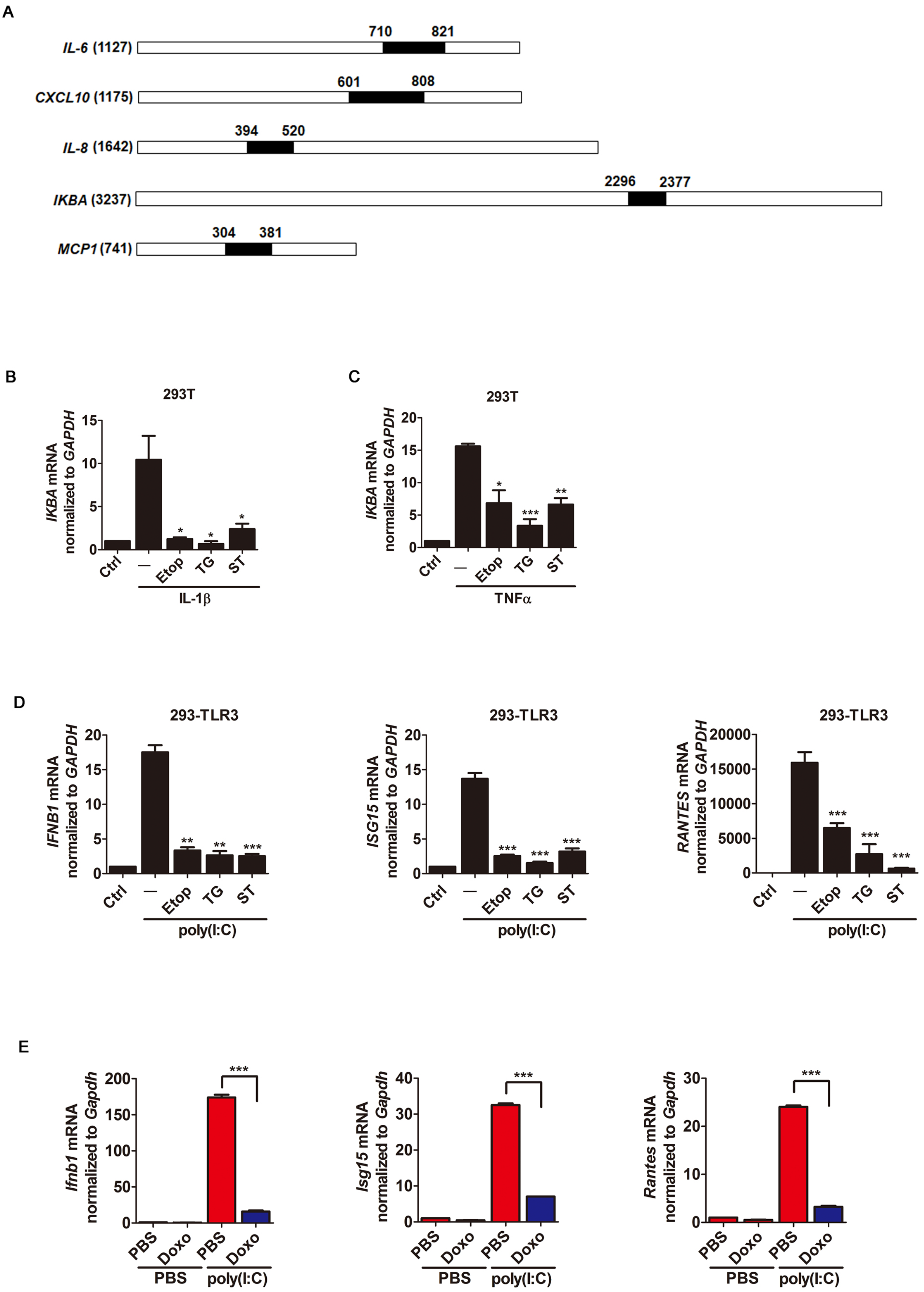
A
IL-6 (1127)
710
821
CXCL10 (1175)
601
808
IL-8 (1642)
394
520
IKBA (3237)
2296
2377
MCP1 (741)
304
381
B
293T
IKBA mRNA normalized to GAPDH
Ctrl
—
Etop
TG
ST
IL-1β
C
293T
IKBA mRNA normalized to GAPDH
Ctrl
—
Etop
TG
ST
TNFα
D
293-TLR3
IFNB1 mRNA normalized to GAPDH
ISG15 mRNA normalized to GAPDH
RANTES mRNA normalized to GAPDH
Ctrl
—
Etop
TG
ST
poly(I:C)
E
Ifnb1 mRNA normalized to Gapdh
Isg15 mRNA normalized to Gapdh
Rantes mRNA normalized to Gapdh
PBS
Doxo
PBS
Doxo
PBS
poly(I:C)

Figure EV2. Apoptosis inhibits cytokine and INF-I signaling pathway. Related to Figure 1.

(**A**) The gene transcript map of regions of IL-6, CXCL10, IL-8, IKBA, MCP1 for qRT-PCR. (**B, C** and **D**) Quantitative RT-PCR analysis of indicated mRNAs in 293T cells or 293-TLR3 cells treated with Etop, TG, or ST in the absence or presence of IL-1β (10 ng/mL) for 4 hr (D), TNFα (10 ng/ml) for 2 hr (E) or poly(I:C) (F). (**E**) Sex- and age-matched mice (n=10) were ip injected of 20 mg/kg doxorubicin (DOX or Doxo) or PBS for 5 days, ip injected with poly(I:C) or PBS for 2 h. *ifnb1*, *Isg15* and *Rantes* mRNAs of heart were analyzed by qRT-PCR.

Data are representative of three independent experiments (means with SEMs). $^*p < 0.05$, $^{**}p < 0.01$, and $^{***}p < 0.001$.

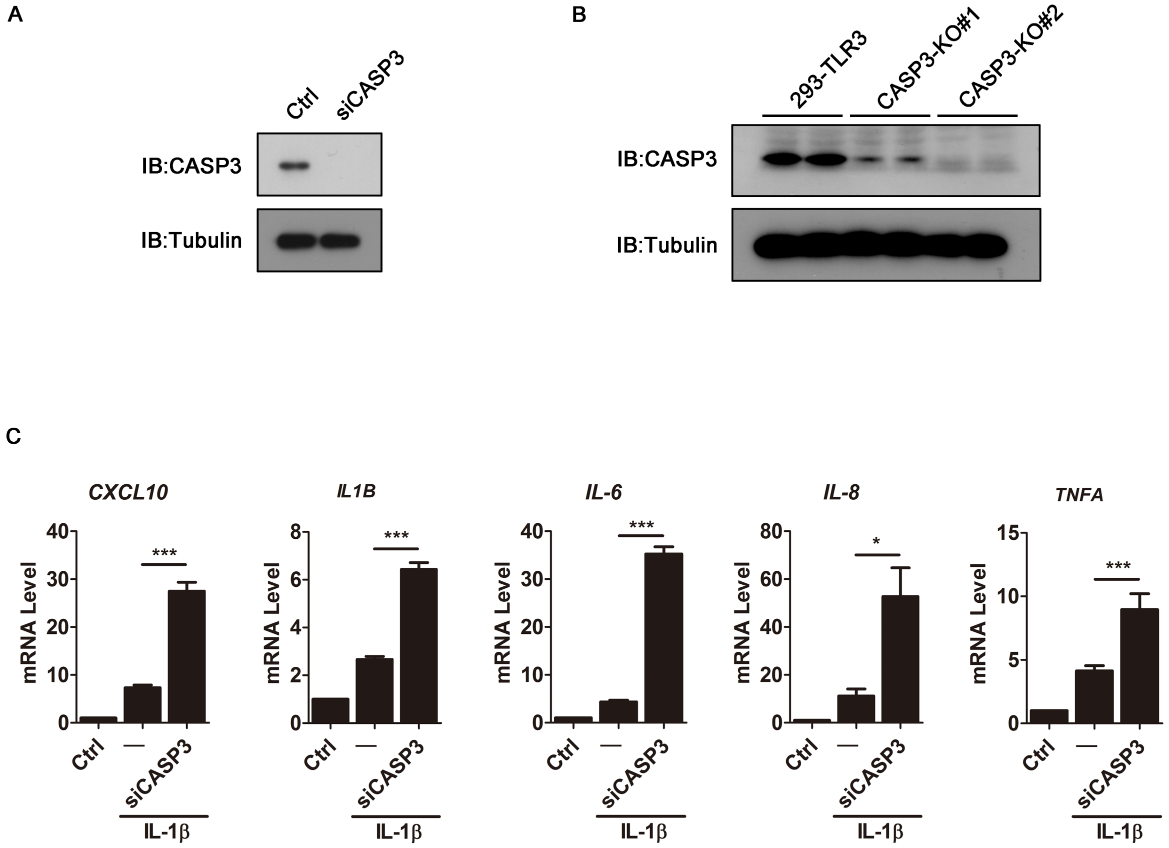

A
Ctrl
siCASP3
IB:CASP3
IB:Tubulin
B
293-TLR3
CASP3-KO#1
CASP3-KO#2
IB:CASP3
IB:Tubulin
C
CXCL10
IL1B
IL-6
IL-8
TNFA
mRNA Level
***
*
Ctrl
—
siCASP3
IL-1β

Figure EV3. The effects of caspase-3 knockdown and knockout. Related to Figure 2

(**A**) 293T cells were transfected with control siRNA or siRNA against caspase-3 for 48 hr, followed by Western blots with indicated antibodies. (**B**) 293-TLR3 cells were transfected with plasmid encoding CRISPR-Cas9 and one of two distinct sgRNAs (#1 and #2) against caspase-3, followed by puromycin treatment and selection. The caspase-3 knockout (KO) effects were determined by Western blots with indicated antibodies. 293-TLR3-CASP3-KO#2 was chosen to be used in Figure 1A. (**C**) Quantitative RT-PCR analysis of *CXCL10*, *IL1B*, *IL-6*, *IL-8* and *TNFA* mRNA in 293T cells transfected with control siRNA or siRNA against caspase-3, and treated with IL-1β as indicated.

Data are representative of three independent experiments (means with SEMs). $*p < 0.05$, $**p < 0.01$, and $***p < 0.001$.

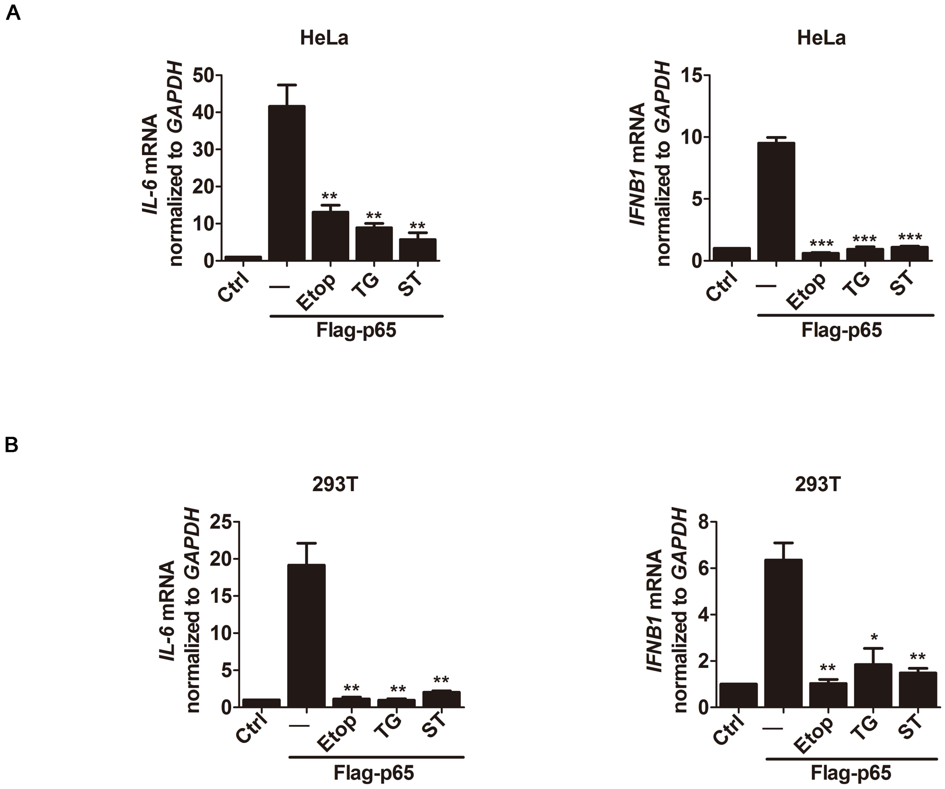
A
HeLa
IL-6 mRNA normalized to GAPDH
50
40
30
20
10
0
Ctrl
—
Etop
TG
ST
Flag-p65
**
**
**
HeLa
IFNB1 mRNA normalized to GAPDH
15
10
5
0
Ctrl
—
Etop
TG
ST
Flag-p65
***
***
***
B
293T
IL-6 mRNA normalized to GAPDH
25
20
15
10
5
0
Ctrl
—
Etop
TG
ST
Flag-p65
**
**
**
293T
IFNB1 mRNA normalized to GAPDH
8
6
4
2
0
Ctrl
—
Etop
TG
ST
Flag-p65
**
*
**

Figure EV4. Caspase-3 downregulates cytokine signaling pathway induced by p65/RelA. Related to Figure 3.

(**A** and **B**) Quantitative RT-PCR analysis of *IL-6* and *IFNB1* mRNA in HeLa and 293T cells transfected with transfected with the Flag-p65 plasmid and treated with Etop, TG, or ST as indicated.

Data are representative of three independent experiments (means with SEMs). $^{*}p < 0.05$, $^{**}p < 0.01$, and $^{***}p < 0.001$.

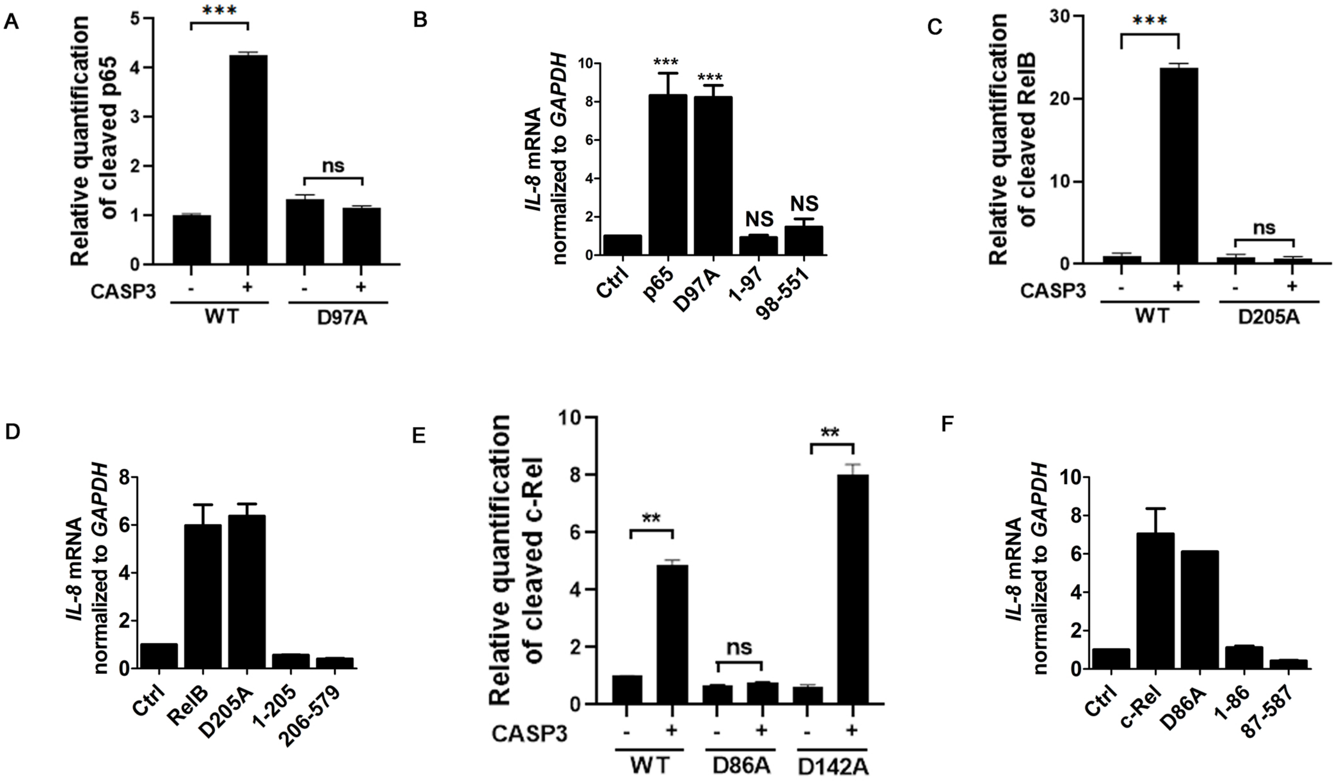
A
Relative quantification of cleaved p65
***
ns
CASP3
- + - +
WT
D97A
B
IL-8 mRNA normalized to GAPDH
***
***
NS
NS
Ctrl
p65
D97A
1-97
98-551
C
Relative quantification of cleaved RelB
***
ns
CASP3
WT
D205A
D
IL-8 mRNA normalized to GAPDH
Ctrl
RelB
D205A
1-205
206-579
E
Relative quantification of cleaved c-Rel
**
ns
**
CASP3
WT
D86A
D142A
F
IL-8 mRNA normalized to GAPDH
Ctrl
c-Rel
D86A
1-86
87-587

Figure EV5. Caspase-3-mediated RelA, RelB and c-Rel cleavage result in their inactivation. Related to Figure 4.

(**A**, **C** and **E**) Relative quantification of indicated protein bands. (**B**, **D** and **F**) Quantitative RT-PCR analysis of *IL-8* mRNA in HEK293T cells transfected with RelA, RelB and c-Rel, as well as theirs mutant or truncations as indicated.

Data are representative of three independent experiments (means with SEMs). $*p < 0.05$, $**p < 0.01$, and $***p < 0.001$.

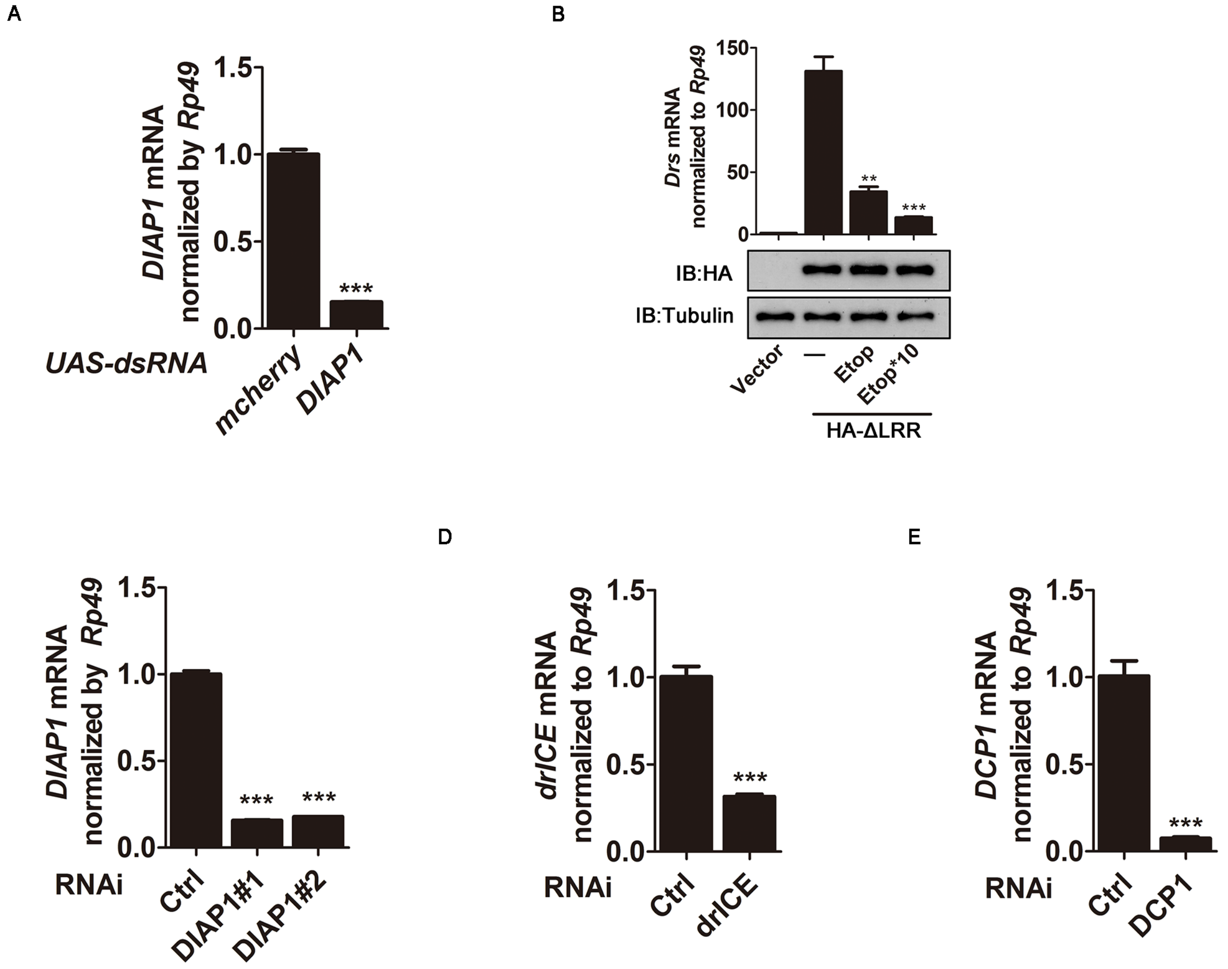
A
DIAP1 mRNA normalized by Rp49
1.5
1.0
0.5
0.0
***
UAS-dsRNA
mcherry
DIAP1
B
Drs mRNA normalized to Rp49
150
100
50
0
**
***
IB:HA
IB:Tubulin
Vector
—
Etop
Etop*10
HA-ΔLRR
C
DIAP1 mRNA normalized by Rp49
1.5
1.0
0.5
0.0
***
***
RNAi
Ctrl
DIAP1#1
DIAP1#2
D
drICE mRNA normalized to Rp49
1.5
1.0
0.5
0.0
***
RNAi
Ctrl
drICE
E
DCP1 mRNA normalized to Rp49
1.5
1.0
0.5
0.0
***
RNAi
Ctrl
DCP1

Figure EV6. The knockdown of DIAP, Cactus, drICE and DCP1 in S2 cells and adult flies. Related to Figure 5.

(**A**) Quantitative RT-PCR analysis of DIAP1 mRNA in adult flies with the indicated genotypes. (**B**) S2 cells were transfected with the HA-TollΔLRR plasmid in the presence or absence of Etop (10 or 100 μM, 24 hr). The samples were subjected to qRT-PCR analysis of Drs mRNA and Western blots with indicated antibodies. (**C**, **D** and **E**) Quantitative RT-PCR analysis of *DIAP1*, *drICE* and *DCP1* mRNA transfected with dsRNA against the indicated genes.

Data are representative of three independent experiments (means with SEMs). $*p < 0.05$, $**p < 0.01$, and $***p < 0.001$.

Table S1. The primers and oligonucleotides used in this study.

primers	Sequence (5' to 3')a,b	Purpose
Flag-DIF For	GGGGTACCTACCCATACGATGTTCCAGATTACGCTTAAATGTTTGAGGAGGCTTTCGG	Construction of plasmid
Flag-DIF Rev	CCCTCGAGTCATTTGAATGGCTGAATTC	
HA-Rpr For	GGGGTACCATGTACCCATACGACGTCCCAGACTACGCTGCAGTGGCATTCTACAT	
HA-Rpr Rev	GCTCTAGATCATTGCGATGGCTTGCGAT	
HA-drICE For	GGGGTACCATGTACCCATACGATGTTCCAGATTACGCTGACGCCACTAACAATGGAGAAT	
HA-drICE Rev	CCCTCGAG TCAAACCCGTCCGGCT	
HA-DCP1 For	GGGGTACCATGTACCCATACGATGTTCCAGATTACGCTGCCAAGGGCTGTACGCCGGAGTCTCT	
HA-DCP1 Rev	CCCTCGAGCTAGCCAGCCTTATTGCCGTTCGGCTTGTCGC	
DIF-D213A For	AAATTCGATCACAAGGACCAGATCGACAAGATC	
DIF-D213A Rev	GATCTTGTCGATCTGGTCCTTGTGATCGAATTT	
IRF3-D72A For	GGGAGGGATAAGCCAGCCCTGCCAACCTGGAAG	
IRF3-D72A Rev	CTTCCAGGTTGGCAGGGCTGGCTTATCCCTCCC	
IRF3-D102A For	AGCAAGGACCCTCACGCCCCACATAAAATCTACGAG	
IRF3-D102A Rev	CTCGTAGATTTTATGTGGGGCGTGAGGGTCCTTGCT	
IRF3-D141A For	CCAGGAAGACATTCTGGCTGAGTTACTGGGTAACA	
IRF3-D141A Rev	TGTTACCCAGTAACTCAGCCAGAATGTCTTCCTGG	
drICE-C211A For	GTTCTTCATACAGGCCGAGCAGGGCGACAGATTG	
drICE-C211A Rev	CAATCTGTCGCCCTGGCAGGCCTGTATGAAGAAC	
DCP1-C196A For	GCTGTTCTTCATCCAAGCCGCGCAGGGCGATCG	
DCP1-C196A Rev	CGATCGCCCTGCGCGGCTTGGATGAAGAACAGC	
DIAP1 dsRNA#1 For	TAATACGACTCACTATAGGCATGAATGCAGTAAATGCGG	Construction of in vitro-transcription templates for preparing dsRNAs for RNAi
DIAP1 dsRNA#1 Rev	TAATACGACTCACTATAGGCAATCGAAGTGCTCTCCTCC	
DIAP1 dsRNA#2 For	TAATACGACTCACTATAGGTGTTTTTCCGTTCGTGTTCA	
DIAP1 dsRNA#2 Rev	TAATACGACTCACTATAGGGCTCCTTTGTTTGCCTGACT	
drICE dsRNA For	*TAATACGACTCACTATAGG*ACTGCCGCTACAAGGACATT	
drIEC dsRNA Rev	*TAATACGACTCACTATAGG*GCGTGCACTGGAATCTTGTA	
DCP1 dsRNA For	TAATACGACTCACTATAGGGCACAAGGACTGCAAGTTGA	
DCP1 dsRNA Rev	TAATACGACTCACTATAGGGTACTTTTTGCCATTGGCGT	

Drosomycin probe For	ATAATTCAAACAGAAATCATTTACCAAGCTCCGTGAGAAC	Construction of in vitro-transcription templates for preparing probes for Northern blots analysis
Drosomycin probe Rev	*TAATACGACTCACTATAGG*AATGTACATTAGTTTTGTTTATTAG	
Rp49 probe Rev	CCGTGAATACTGTGGTGAAATTGCC	
Rp49 probe Rev	*TAATACGACTCACTATAGG*TTTTTTTTTCACTTTTAACGTTTCA	
Rp49 qRT For	AGCATACAGGCCCAAGATCG	Primers used for qRT-PCR
Rp49 qRT Rev	TGTTGTCGATACCCTTGGGC	
DIAP1 qRT For	CCCCAGTATCCCGAATACGC	
DIAP1 qRT Rev	TCTGTTTCAGGTTCCTCGGC	
Diptericin qRT For	ACCGCAGTACCCACTCAATC	
Diptericin qRT Rev	CCCAAGTGCTGTCCATATCC	
Drosomycin qRT For	CGTGAGAACCTTTTCCAATATGATG	
Drosomycin qRT Rev	TCCCAGGACCACCAGCA T	
drICE qRT For	ATGGACGCCACTAACAATGGA	
drICE qRT Rev	AGGGCATCTGTGTGATCGTTG	
DCP1 qRT For	GGAAAATCGGGGCAGCTTTAT	
DCP1 qRT Rev	CATTCGAGCCACAAACTTGTTG	
IFNB1 qRT For	AGGACAGGA TGAACTTTGAC	
IFNB1 qRT Rev	TGATAGACATTAGCCAGGAG	
GAPDH qRT For	A TGACA TCAAGAAGGTGGTG	
GAPDH qRT Rev	CATACCAGGAAATGAGCTTG	
CXCL10 qRT For	GCTCTACTGAGGTGCTATGTTC	
CXCL10 qRT Rev	GGAGGATGGCAGTGGAAGTC	
Il6 qRT For	TTCCA TCCAGTTGCCTTCTTG	
Il6 qRT Rev	AATTAAGCCTCCGACTTGTGAA	
IKBA qRT For	GAGTGAGGATGAGGAGAG	
IKBA qRT Rev	AGTCTGTGAACTCCTTCG	
MCP1 qRT For	AGAAGTGGGTTCAGGATT	
MCP1 qRT Rev	GAACACTCACTCCACAAC	
RANTES qRT For	TACACCAGTGGCAAGTGCTC	
RANTES qRT Rev	ACACACTTGGCGGTTCTTTC	

ISG15 qRT For	GAGAGGCAGCGAACTCATCTT	
ISG15 qRT Rev	CCAGCA TCTTCACCGTCAGG	
TNFA qRT For	AAGAGATGTGGCAAGAGA	
TNFA qRT Rev	TTCATTCATTCATTCATTCATTCA	
IL-6 qRT For	ACCTCAGATTGTTGTTGT	
IL-6 qRT Rev	GTCCTAACGCTCATACTT	
IL -8 qRT For	AATTCATTCTCTGTGGTATC	
IL -8 qRT Rev	CCAGGAATCTTGTATTGC	
Gapdh qRT For	A TGGTGAAGGTCGGTGTGAA	
Gapdh qRT Rev	CGCTCCTGGAAGA TGGTGA T	
Isg15 qRT For	CCTCTGAGCA TCCTGGTGAG	
Isg15 qRT Rev	ACTGGTCTTCGTGGACTTGTT	
Cxcl10 qRT For	TCAGGCTCGTCAGTTCTAAGTT	
Cxcl10 qRT Rev	GATGGTGGTTAAGTTCGTGCTT	
Ccl5 qRT For	AGGACTCTGAGACAGCACA T	
Ccl5 qRT Rev	GCAGTGAGGA TGA TGGTGAG	
Ifnb1 qRT For	CCGAGCAGAGA TCTTCAGGAA	
Ifnb1 qRT Rev	CCTGCAACCACCACTCATTCT	
si-h-caspase 3_001	AGTGAAGCAAATCAGAAAC	siRNA target sequences
si-h-caspase 3_002	TGGATTATCCTGAGATGGG	
si-m-Casp3_001	CGAAAGAACTGTACTTTTA	
si-m-Casp3_002	CAACGGAATTCGAGTCCTT	
